# Routine Immunization Programs for Children during the COVID-19 Pandemic in Ecuador, 2020—Hidden Effects, Predictable Consequences

**DOI:** 10.3390/vaccines10060857

**Published:** 2022-05-27

**Authors:** Gianina Lizeth Suárez-Rodríguez, José Salazar-Loor, Jackson Rivas-Condo, Alfonso J. Rodríguez-Morales, Juan-Carlos Navarro, José Rubén Ramírez-Iglesias

**Affiliations:** 1 Research Group of Emerging and Neglected Diseases, Ecoepidemiology and Biodiversity, Health Sciences Faculty, Universidad Internacional SEK (UISEK), Quito 170120, Ecuador; gianina.suarez@uisek.edu.ec (G.L.S.-R.); jose.salazar@uisek.edu.ec (J.S.-L.); juancarlos.navarro@uisek.edu.ec (J.-C.N.); 2 Program of Master in Biomedicine, Health Sciences Faculty, Universidad Internacional SEK (UISEK), Quito 170120, Ecuador; 3 Faculty of Engineering and Applied Sciences, Universidad Internacional SEK (UISEK), Quito 170120, Ecuador; 4 Secretaría de Salud del Distrito Metropolitano de Quito, Quito 170136, Ecuador; jackrivas88@hotmail.com; 5 Grupo de Investigación Biomedicina, Faculty of Medicine, Fundación Universitaria Autónoma de las Américas, Pereira 660001, Risaralda, Colombia; alfonso.rodriguez@uam.edu.co; 6 Program of Master in Clinical Epidemiology and Biostatistics, Universidad Científica del Sur, Lima 150142, Peru

**Keywords:** routine immunization, vaccination, infants, pandemic, COVID19, preventable diseases

## Abstract

The COVID-19 pandemic has led to a global disruption of several services, including routine immunizations. This effect has been described in several countries, but there are few detailed studies in Latin America and no reports in Ecuador. Therefore, this work aims to quantify the reduction in routine immunizations for infants during the 2020 COVID-19 pandemic in Ecuador. 2018, 2019, and 2020 data were obtained from the Ministry of Health, Ecuador. The number of doses and the extent of immunization coverage was descriptively compared for four vaccines: rotavirus (ROTA), poliovirus (PV), pneumococcal (PCV), and pentavalent (PENTA) vaccines. There was no significant difference in doses applied during the 2018 and 2019 years. However, a significant (*p* < 0.05) drop of 137,000 delivered doses was observed in 2020 compared to the pre-pandemic years. Reductions in the percentage of coverage were more pronounced for the PENTA vaccine (17.7%), followed by PV (16.4%), ROTA (12%), and PCV vaccines (10.7%). Spatial analysis shows a severe impact on vaccination coverage on provinces from the Coast and Highland regions of the country. The pandemic has significantly impacted the immunization programs for infants across Ecuador. This retrospective analysis shows an urgent need to protect vulnerable zones and populations during public health emergencies.

## 1. Introduction

An outbreak of atypical pneumonia was reported in December 2019 in Wuhan, China, caused by the novel coronavirus SARS-CoV-2 which produced the coronavirus disease in 2019 (COVID-19) [1]. On 11 March, the WHO declared the COVID-19 a pandemic [2] and, by October 2020, the virus had spread all over the world [3]. In this scenario, and with no treatments or vaccines available during the initial months of the pandemic, several non-pharmaceutical intervention measures were taken to control the spread of SARS-CoV-2, mitigate its effects on populations, and reduce the burden on healthcare systems. These measures included the everyday use of personal protective equipment, diligent hand hygiene practices, social distancing, stay-at-home orders, and movement restrictions at several geographical levels [4,5]. However, disruption in health services, particularly in low-income and middle-income countries (LMIC), has been reported, including the redirection of services to the COVID-19 response, healthcare facility closures, negative impacts on medical supply chains, and interruptions to routine immunization programs [6]. Vaccination is one of the most successful measures to reduce infant morbidity and mortality and confers protection across childhood. Securing programs for timely immunizations is critical to preventing outbreaks of vaccine-preventable diseases (VPD) and to decreasing the risk of re-emerging diseases in countries susceptible to natural disasters [7,8]. Despite the worldwide efforts to maintain essential services related to healthcare settings, several LMIC and some high-income countries reported severe disruptions in their routine immunization programs for infants in 2020, with a significant decline in the number of doses administered and the extent of vaccination coverage in the USA, the United Kingdom, Spain, Pakistan, Sierra Leone, and the region of South-East Asia and the Western Pacific, among others [9,10,11,12,13,14,15]. Similarly, the Global Alliance for Vaccines and Immunization (GAVI) reported that 13.5 million people in the least-developed countries of the world would not be protected against diseases such as measles, polio, and human papillomavirus [16]. Multiple factors such as a concern about leaving home and being exposed to COVID-19 as well as interruptions in transport systems have been associated with delayed immunizations during the pandemic [17,18].

The first case of COVID-19 in the South American country of Ecuador was reported on 29 February 2020. Subsequently, a state of national emergency was declared on 11 March, with measures implemented for mitigating the transmission of SARS-CoV-2 at the population level [19]. These measures consisted of pedestrian and vehicle mobility restrictions, the suspension of mass events, national and international flight disruptions, the closing of borders, stay-at-home orders, and remote working for non-essential services [20]. Most of these directions were nationally reduced by October–November 2020, although some measures were maintained according to the situation of specific provinces and cities. The Ecuadorian immunization program for infants (children under one year) is comprised of meningococcal, hepatitis B, rotavirus, poliovirus, pneumococcal, pentavalent (diphtheria, pertussis, tetanus, hep B, Hemophilus), and influenza vaccines [21]. However, there are few studies of the impact of the COVID-19 pandemic on routine immunizations for infants in South America, and no detailed studies about the variations in the vaccine-administered doses for children in Ecuador. The progressively adopted measures for controlling transmission of SARS-CoV-2 in Ecuador represent an important context to analyze the routine vaccination process. Therefore, this study aims to evaluate the effects of the COVID-19 mitigation measures on routine immunization programs for infants in Ecuador.

## 2. Materials and Methods

### 2.1. Study Population and Databases

Ecuador is classified as an LMIC [22] and its territory comprises 24 provinces within 3 regions. The coastal region, consisting of Esmeraldas, Manabi, Los Ríos, Santa Elena, Guayas, Santo Domingo de los Tsáchilas and El Oro; the highlands region, consisting of Azuay, Bolívar, Cañar, Carchi, Cotopaxi, Chimborazo, Imbabura, Loja, Pichincha and Tungurahua; and the Amazon region, consisting of Morona Santiago, Napo, Orellana, Pastaza, Sucumbíos and Zamora Chinchipe, and the Insular region comprising Galapagos islands.

The databases of all provinces were provided and approved for use under the authorizations MSP-DNEPC-2021-0003-O and MSP-DNEAIS-2021-0069-O by the National Direction of Statistics and Health Information Analysis of the Ministry of Health (MSP), Ecuador, which is the only department in charge that centralizes national public registries. Vaccination campaigns led by the Ministry of Health represent nearly 95% of the doses administered in the country. These immunization data contain information about vaccines exclusively. Doses applied, years, months, general geographical locations, and no personal information. A total of four types of vaccines within the Ecuadorian routine immunization programs for infants under one year were compared: the ROTA, PV, PCV, and PENTA vaccines for the pre-pandemic years 2018–2019 and for the initial year of the COVID-19 pandemic in 2020. The national program recommends applying two doses for the ROTA vaccine at 2 and 4 months and three doses for the PV, PCV, and PENTA vaccines at 2, 4, and 6 months.

The data were compared from March–at the beginning of the lockdown declaration in Ecuador–to December, for the 2018 and 2020 years. Information about the population of infants for each region and province was also obtained from the MSP (Table 1). The vaccination coverage calculated in this study is the proportion of children in the Region receiving the recommended vaccines [23]. This indicator is the result of the ratio between the number of doses administered with a specific antigen and the number of infants in a locality.

Due to the absence of personal information in the dataset or direct intervention on the aimed population, no bioethical approval was required.

### 2.2. Statistical and Spatial Analysis

We used the Kruskal Wallis test, which is a nonparametric approach, to compare three or more groups by a dependent variable that is measured on at least an ordinal level. Here we compare the doses administered and the extent of vaccination coverage from 2018, 2019, and 2020, at national and provincial scales. The variation in the vaccination coverage between the analyzed years was calculated using the following formula: vaccination coverage percentage = (Year 1 Coverage − Year 2 Coverage/Year 1 Coverage) × 100. In the case of doses administered, the variation was calculated as the difference between two years. A specific analysis was performed using the data for the doses applied during April, a month with strict measures imposed by the local government, and November, which saw a flexibilization in the adopted restrictions. The differences were significant, with *p* < 0.05. The IBM SPSS v25 (Armonk, NY, USA) software was used for graphs and statistical tests. To explore possible differences in the influence of the COVID-19 pandemic context on the vaccination process in the Ecuadorian territory, a spatial clustering analysis was performed to establish the potential heterogeneity of vaccination coverage in all the evaluated years at the provincial and cantonal levels, using the Global Moran’s I and Getis–Ord Gi statistics [24,25].

The Global Moran’s I indicate the degree to which points are like their spatial neighbors. Negative values indicate strong negative autocorrelation (high spatial dispersion), values higher than two indicate a positive correlation or clustering, and data between 0 and 2 are considered random patterns. The Getis–Ord Gi was used for the hotspot and clustering analysis, where cold spots are low values clustered in areas and hot spots are high values clustered in a location. The statistics were obtained using the ArcGIS Pro 10.8 software. Additionally, to evaluate the trend in the vaccination process across 2018–2020, and to detect specific breakpoints in immunizations administered in Ecuador, a Joinpoint analysis [26] was performed with the number of last vaccination doses administered for all the antigens studied. The Joinpoint Regression Software v4.9.1.0. was used for this trend analysis.

## 3. Results

### 3.1. Population of Study and Changes in the National Administered Doses and Vaccination Coverage

The infant population of the pre-pandemic and pandemic years was analyzed to determine if any alteration in the national vaccination program was due to changes in the newborn registration. According to the MSP data, the number of infants registered was 332,505 (2018), 331,773 (2019), and 330,970 (2020), with no statistical differences between the analyzed years.

**Figure 1:**
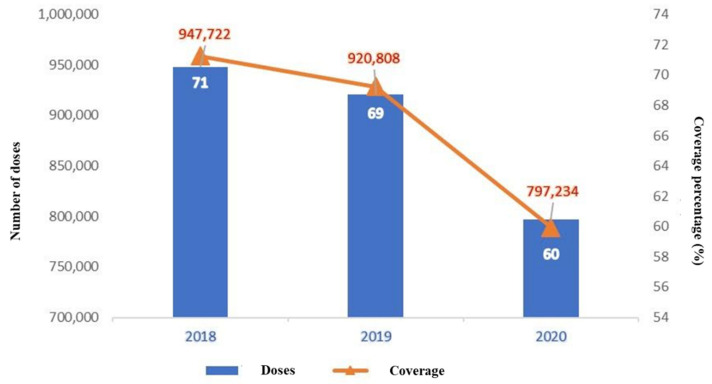
Administered doses and vaccination coverage percentages for the ROT, PV, PCV, and PENTA vaccines in Ecuador. The graph shows the total number of final doses and the coverage: second dose for ROT, and third dose for the rest of the antigens.

The total numbers of the last vaccine doses administered in Ecuador were 947,722 and 920,808, during 2018 and 2019, with a mean for pre-pandemic years of 934,265. In 2020, the number dropped to 797,234, with a significant reduction of 137,031 and a variation of 14% in the vaccination coverage percentage compared to the 2018/2019 mean for the last doses at the national scale (Figure 1). The detailed last administered doses and vaccination coverage percentages are shown in Table 2, where the pre-pandemic years presented similar behavior.

**Figure 2:**
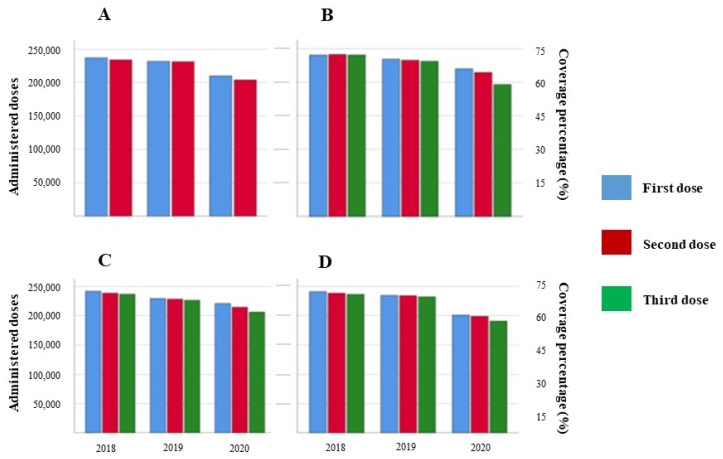
Changes in administered vaccination doses and coverage percentages. The graph shows the detailed number of every dose administered and the vaccination coverage in Ecuador during the three analyzed years. Panels: (**A**) ROTA; (**B**) PV; (**C**) PCV; (**D**) PENTA.

However, 2020 shows a significant disruption for all four antigens evaluated with mean reduction values of 43,476 (17.67%), 39,389 (16.38%), 28,672 (12.01%), and 25,493 (10.65%) for the PENTA, PV, ROTA, and PCV vaccines, respectively (Table 2). This reduction shown in 2020 is observed for the three mandatory doses for PENTA, PV, PCV, and the two doses in the case of the ROTA vaccine (Figure 2, panels A; B; C; D). Interestingly, the last doses were the most affected for all the antigens. The third administered dose and vaccination coverage percentage for PENTA (190,796/57.65%) and PCV (206,205/62.3%) were the most and least affected vaccines analyzed in this study, respectively.

In general, we registered an average reduction of 14.18% of vaccination coverage for the antigens evaluated here.

### 3.2. Spatial Pattern Analysis of Vaccination Coverage

Due to the statistical differences displayed by the doses administered and immunization coverage percentages in 2020 compared to 2018–2019, we decided to perform a spatial analysis to detect possible clustering areas and specific patterns for the vaccination coverage rates in 2020.

**Figure 3:**
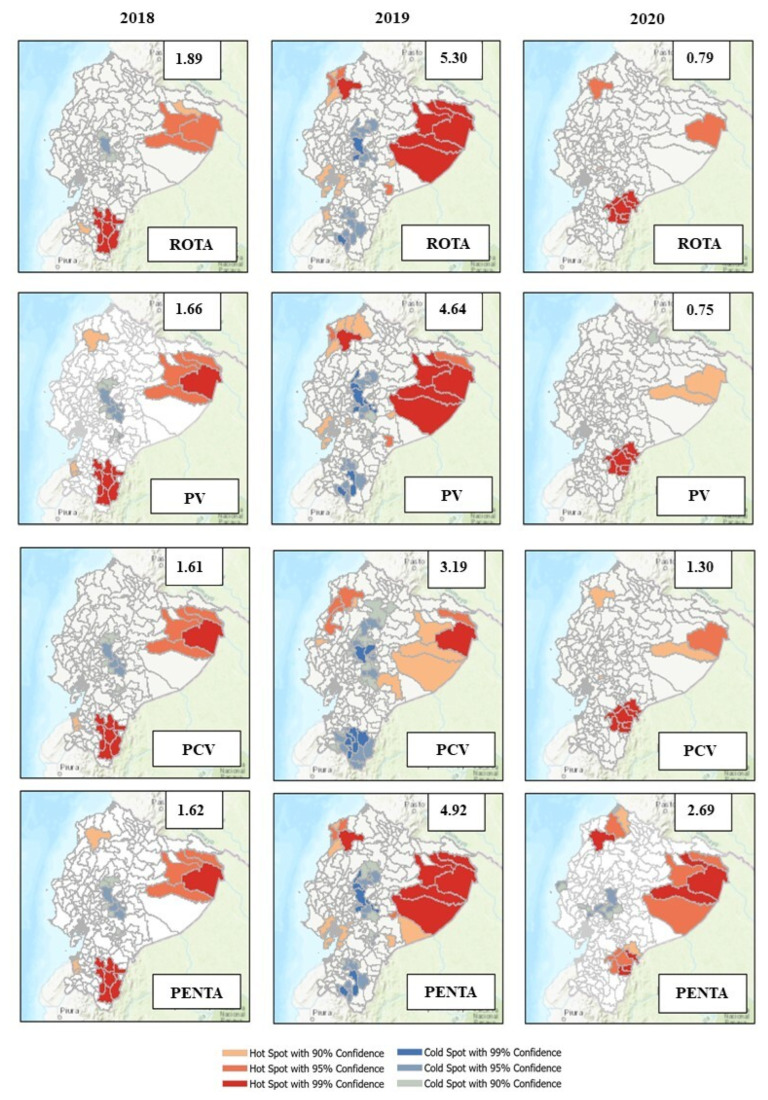
Spatial analysis of vaccination coverage rates for infants in 2018, 2019, and 2020 in Ecuador. The maps show patterns of high (red) and low (blue) vaccination coverage areas compared to their neighboring locations. Numbers in the superior square indicate the Global Moran’s I index, calculated using the coverage of the last dose of every vaccine applied.

The interannual analysis shows high and random clustering values for the coverage of evaluated vaccines in the pre-pandemic years, indicated by the Global Moran’s I index. Changes in the pattern of high vaccination coverage for all the antigens in 2020 were detected, especially in the Orellana and Pastaza provinces, located in the eastern region of the country (Figure 3). The average coverage registered for these provinces was 97.48% and 91.17% in 2018–2019, which were superior values compared to the 86.42% and 79.03%, registered in 2020. Similarly, the Esmeralda province, located in northwestern Ecuador, presents changes in the clustering of hot spots, with average coverage values of 93.98% in 2018–19 and 80.03% in 2020. The intra-annual analysis of 2020 indicates highly dispersed data regarding the vaccination coverage values for all types of antigens, and a few hot and cold spots of clustering across the Ecuadorian territory, except for the analysis of the PENTA vaccine (Figure 3). This general lack of clustering, observed in the maps as areas without color, indicates that immunizations with ROTA, PV, and PCV vaccines were similarly affected in the cantons and provinces of the territory. This result is also indicated by the Global Moran’s I index, whose values oscillated between 0 and 2 for these antigens.

Cold spots in the 2020 maps indicate low vaccination coverage percentages for these three antigens in the Guayas province, with a 76.25% vaccination coverage. In the case of the PENTA vaccine, the cold spots include the Los Rios, Manabí, Bolivar, and Chimborazo provinces, with coverage values of 74.54%, 78.80%, 67.85%, and 69.18%, respectively. The vaccination coverage values for these five provinces in the pre-pandemic years were 88.51%, 84.51%, 90.04%, 68.58%, and 73.18%.

The insular region was not included in this analysis due to the absence of neighboring counterparts. The general percentages of vaccination coverage in 2020 calculated for the Amazon, Coast, Highland, and Insular regions were 84.53%, 75.74%, 70.36%, and 69.33%, respectively.

### 3.3. Yearly Trend Analysis and Administered Vaccine Doses during April and November 2018–2020

**Figure 4:**
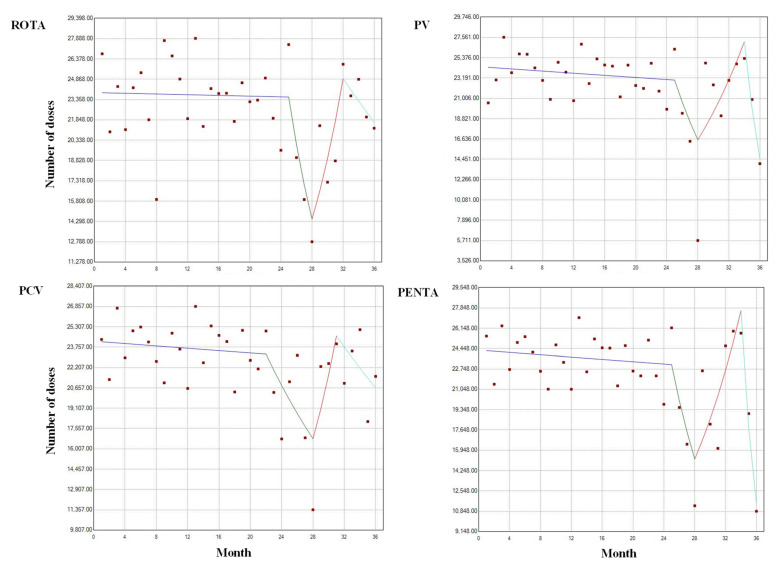
Yearly Joinpoint analysis of immunizations administered for infants in Ecuador. The four graphs show the number of the last doses administered of rotavirus, poliovirus, pneumococcal, and pentavalent vaccines. Months (January to December) and years (2018 to 2020) covered: 0–12, 2018; 13–24, 2019; 25–36, 2020.

The Joinpoint analysis used to detect specific breakpoints and changes related to immunizations is displayed in Figure 4, which shows all antigens with a stable trend, around 22,000 to 25,000 immunizations, in the administered doses from month 0 (January 2018) to nearly month 26 (February 2020). The only exception was the PCV vaccine, for which the breakpoint begins on month 23 (November 2019). The lowest point in the vaccination trend is observed in month 28 (April 2020) for the four antigens, with doses oscillating between 14,000 and 16,000 immunizations. Subsequently, all administered doses presented an increasing trend with higher doses administered around months 32 (August 2020) to 34 (November 2020). The PENTA and PV vaccines presented a marked decreasing trend, showing new low immunization levels between 10,000 and 14,000 doses administered, respectively.

The following specific analysis aimed to determine the possible influence of the government measures against the COVID-19 pandemic imposed during April 2020 on the national vaccination process and its subsequent flexibilization in November of the same year. During April, the pre-pandemic years show similar numbers, between 21,000 and 24,000 administered doses, with the highest variation around 2600 for the PCV vaccine. In 2020, the significant difference with 2018–2019 in the last applied dose was maintained for all the antigens. In this case, immunization with the PV vaccine was the most affected, followed by the PCV, PENTA, and ROTA antigens, with reduction means of 18,468, 12,449, 12,310, and 9672, respectively (Table 3).

However, there was no statistical difference between November 2020 and the two other evaluated years, with around 20,000 administered doses for all the antigens in the initial year of the pandemic (Table 4). Despite this apparent recovery, the mean drops of applied doses oscillated between 1365 for ROTA and 3865 for PCV as the least and most affected administered vaccines.

## 4. Discussion

In this study, we analyzed the influence of the COVID-19 pandemic on routine immunization programs for infants during 2020 in Ecuador. The size of the population to be vaccinated (infants under one year of age) did not vary significantly in the three years analyzed, including 2020, the year of the pandemic that was evaluated; therefore, the reduction in vaccination coverage in this age group in 2020 might not have been the consequence of a reduction of infants to be immunized but another independent effect. Overall, the data show a severe disruption for the four antigens evaluated at the national scale, for both administered doses and coverage percentages. The reduction of the doses in 2020 was 137,031, about 14% fewer vaccines administered compared to 2018/2019.

Similarly, we described the national percentage coverage reduction, with the PENTA vaccine as the most affected antigen within the routine program. These values indicate that a specific population of infants missed the ROTA, PV, PCV, and PENTA vaccinations during the months between March and December of 2020.

To the best of our knowledge, this is the first detailed research analyzing the impact of the pandemic on infant immunization in Ecuador, and it is among only few studies published in Latin America. A global study estimating the disruption in routine immunizations in several regions reported a general 7.7% and 7.9% reduction for the measles and diphtheria-tetanus-pertussis (DTP3) vaccines [27]; the last one includes three out of the five antigens presented in the PENTA vaccine applied in Ecuador. Specifically, a 6.6% reduction for the DTP3 coverage is described in the Latin American region and 6.7% in the case of Ecuador [27].

Our reported values suggest a more drastically affected vaccination process and indicate the importance of measuring the extent of the disruption of immunizations, especially in LMIC countries. The benefits of vaccination across childhood are only guaranteed if the appropriate immunization schedule is completed at the correct time [8]. However, the vaccination coverage values of the last doses for all the antigens were the most reduced in 2020. Although there is no direct explanation for these results, this may be related to several reasons, such as the misconception that good immunity can be achieved by partial immunization, general parental choices, or even using alternative methods, as determined in other studies [28,29].

Although disruptions in routine immunization have been described in several countries and regions of the world, the reduced levels of administered immunizations could be different depending on the province, canton, or subdistrict evaluated in a specific country. The lack of clustering observed in the intra-annual spatial analysis performed for Ecuador suggests that the vaccination coverage was generally affected across the territory. Despite this, specific cold spots were determined in the Guayas province for all the antigens. In fact, the cities in this coastal province presented the highest morbidity and mortality rates during the initial months of the pandemic [30], which lead to the tightening of COVID-19 related domestic measures to control the transmission in this and other provinces [31].

Moreover, the disruption of the PENTA vaccine was more drastic for several provinces of the Coast and Highlands regions of the territory. Conversely, several provinces in the Amazon region presented hot spots, suggesting a high vaccination coverage compared to the other two regions. Nevertheless, it is essential to highlight that the population of the Amazon provinces is significantly less (739,814) compared to the Coast (7,236,822) and the Highlands (6,449,355) regions [32]. Similarly, the specific population behavior of each province and the compliance with the generally adopted measures could be responsible for the differences detected in the immunization processes described in this section. This context and other socio-cultural factors could have influenced the pattern of better vaccination coverage observed in the Amazon area.

Although the Galapagos Islands were not included in this analysis, this region showed the lowest vaccination coverage in this study. The COVID-19 pandemic could be worsened in this area due to its being located outside of the mainland and problems related to shortages and disruptions of medical supply chains, which also affected the global vaccine distribution for routine immunizations [6]. Other studies carried out in Sierra Leone, Pakistan [11], and the Dominican Republic [33] highlighted the importance of detecting vulnerable zones to the impact of immunization programs, which are usually rural areas and subnational locations where inequities affect access to vaccination. Based on this, every country should evaluate its performance during this pandemic to strengthen its public health services and avoid possible outbreaks of several VPDs such as polio, whose eradication may be delayed in several parts of the world due to the pandemic in the current context [34], and measles, which is the main threat due to its high reproduction number [35,36].

Like several countries, Ecuador adopted non-pharmaceutical measures to mitigate viral transmission among its population. The measures were appointed colors according to the level of mobilization restrictions: red for the highest level of restriction, imposed from March 2020 and mainly associated with the movement of vehicles and persons, and green for more flexible measures from October to December 2020. The trend analysis showed similar behavior in the immunizations applied during 2018, and in 2019 until the declaration of the COVID-19 pandemic, in March 2020. Subsequently, the trend indicates a severe decrease in immunizations, which is in concordance with the red stage in April 2020, when the national last administered vaccination doses were significantly lower than pre-pandemic years.

Mobilization restriction, national lockdowns, and stay-at-home orders may be related to these results, although other social factors could be playing specific roles. Surveys applied to parents and caretakers indicate that the fear of contracting the virus, the overburdened health care systems of the countries, the shortage of vaccines, and the lack of protective equipment are among the alternative reasons for the disruption of the vaccination described in various studies [14,15,17,37]. Interestingly, in November 2020, with the green stage implemented, the number of doses administered is similar to 2018/2019, indicating a similar vaccination process for the three evaluated years.

However, it is difficult to suggest a recovery in the the vaccination doses administered in November 2020 due to the overall reduction of immunizations, calculated with the general data from March to December 2020. Similarly, the last doses applied of some antigens such as the PCV and PENTA vaccines presented a decrease towards December 2020. Although no special measures were imposed during this month, this may be related to a shortage in vaccine supplies in the country, an effect that has been reported in other works [6]. Nevertheless, the increasing number of immunizations applied in the last months of 2020 is a behavior observed in several countries and regional studies [27,38].

Based on this scenario, implementing tailored catch-up strategies for unvaccinated children is imperative to prevent possible outbreaks of these diseases, especially when returning to normal activities. Some of the recommendations listed in several studies are recognizing the most affected zones, contacting and identifying those who missed specific immunizations, implementing social media campaigns for the general public, and creating opportunities for vaccination services [12,14].

For this study, we used the best-verified source of information in the country, which was obtained from the Ecuadorian MSP. However, we must emphasize that this data comes from the periodic official vaccination report carried out by each health establishment in the country. Based on this fact, the main limitations in this study are related to the intrinsic bias in the public data, which may contain gaps or erroneous entries. Moreover, it is necessary to clarify that the MSP does not report data from the private sector, which only allows for the analysis of data from public health institutions. Additionally, our approach was mainly based on the number of administered doses to the total yearly population of infants, due to the lack of data supplied and authorized for use by the MSP about this population for specific months of the evaluated years. Based on the design of this study, certain confounding factors remained unadjusted. Thus, we can only hypothesize about the possible reasons for the vaccination disruption in the country and associate the data with the policy restriction during 2020.

## 5. Conclusions

In conclusion, the COVID-19 pandemic caused unprecedented interruptions in vaccine delivery in several countries worldwide. For example, our study reports that there was a reduction in the number of vaccine doses in Ecuador and the vaccination coverage for children under one year of age for the analyzed vaccines (ROTA, PV, PCV, and PENTA). This scenario could be explained by the imminent fear of contagion and the public health measures implemented to mitigate the direct effects of the pandemic. In this sense, the COVID-19 pandemic has disrupted essential health care services around the globe. As a result, several countries with high mortality and morbidity rates due to COVID-19 are still unable to recover from the direct effects of the pandemic. Therefore, Ecuador must restore the systematic efforts to ensure the fulfilment of its childhood immunization schedule. Otherwise, this could initiate the spread of vaccine-preventable diseases in children.

## Figures and Tables

**Table 1:** Total population of infants from the 24 Ecuadorian provinces.

Region	Province	Year		
		2018	2019	2020
Coast	Esmeraldas	13,382	13,293	13,211
	Manabí	29,803	29,499	29,207
	Los Ríos	18,982	18,897	18,888
	Santa Elena	8772	8834	8897
	Guayas	79,639	79,543	79,535
	Santo Domingo	10,532	10,535	10,537
	El Oro	12,597	12,526	12,464
	Subtotal	173,707	173,127	172,739
Highlands	Azuay	15,962	15,943	15,700
	Bolívar	4387	4338	4223
	Cañar	5670	5680	5660
	Carchi	3280	3258	3236
	Cotopaxi	10,408	10,355	10,304
	Chimborazo	10,005	9863	9762
	Imbabura	9202	9173	9141
	Loja	10,031	9978	9,923
	Pichincha	56,493	56,768	57,062
	Tungurahua	10,207	10,159	10,111
	Subtotal	135,645	135,515	135,122
Amazon	Morona Santiago	4886	4865	4842
	Napo	3320	3341	3361
	Orellana	3952	3883	3821
	Pastaza	2618	2639	2659
	Sucumbíos	4920	4940	4958
	Zamora Chinchipe	2840	2839	2837
	Subtotal	22,536	22,498	22,478
Insular	Galápagos	617	624	631
	Total	332,505	331,773	330,970

**Table 2:** Variation of last administered vaccination doses and coverage percentage for infants in Ecuador.

Vaccine	Number of Last Doses (Coverage Percentage)			Variation 2018–2019	*p*-Value 2018–2019	Variation 2018–2020	*p*-Value 2018–2020	Variation 2019–2020	*p*-Value 2019–2020	Variation Pre-Pandemic Years–2020
	2018	2019	2020							
ROTA	234,000 (70.37)	231,167 (69.68)	203,911 (61.61)	2833 (0.98)	0.617	30,089 (12.45)	0.000	27,256 (11.58)	0.000	28,672 (12.01)
PV	240,473 (72.32)	230,950 (69.61)	196,322 (59.32)	9523 (3.75)	0.412	44,151 (17.98)	0.000	34,628 (14.78)	0.000	39,389 (16.38)
PCV	236,881 (71.24)	226,515 (68.27)	206,205 (62.3)	10,366 (4.17)	0.166	30,676 (12.55)	0.000	20,310 (8.74)	0.010	25,493 (10.65)
PENTA	236,368 (71.09)	232,176 (68.98)	190,796 (57.65)	4192 (2.97)	0.762	45,572 (18.91)	0.000	41,380 (16.43)	0.000	43,476 (17.67)

**Table 3:** Last administered vaccination doses for infants during April 2018–2020 in Ecuador.

Vaccine	Administered Doses			Variation 2018–2019	*p*-Value 2018–2019	Variation 2018–2020	*p*-Value 2018–2020	Variation 2019–2020	*p*-Value 2019–2020	Variation PrePandemic Years–2020
	2018	2019	2020							
ROTA	21,121	23,802	12,789	2681	0.304	8332	0.000	11,013	0.000	9,672
PV	23,770	24,590	5712	820	0.811	18,058	0.000	18,878	0.000	18,468
PCV	22,956	24,656	11,357	1700	0.582	11,599	0.000	13,299	0.000	12,449
PENTA	22,712	24,512	11,302	1800	0.474	11,410	0.000	13,210	0.000	12,310

**Table 4:** Last administered vaccination doses for infants during November 2018–2020 in Ecuador.

Vaccine	Administered Doses			Variation 2018–2019	*p*-Value 2018–2019	Variation 2018–2020	*p*-Value 2018–2020	Variation 2019–2020	*p*-Value 2019–2020	Variation PrePandemic Years–2020
	2018	2019	2020							
ROTA	24,882	21,968	22,060	2914	0.689	2822	0.689	92	0.689	1365
PV	23,831	21,783	20,892	2048	0.548	2939	0.548	891	0.548	1915
PCV	23,603	20,316	18,084	3287	0.167	5519	0.167	2232	0.167	3875
PENTA	23,310	22,174	19,017	1136	0.051	4293	0.051	3157	0.051	3725

## Data Availability

Restrictions apply to the availability of these data. Data was obtained from the National Direction of Statistics and Health Information Analysis of the Ministry of Health (MSP) under the authorizations MSP-DNEPC-2021-0003-O and MSP-DNEAIS-2021-0069-O, and are available from the authors with the permission of the MSP.
